# Profiling Combat Sports Athletes: Competitive History and Outcomes According to Sports Type and Current Level of Competition

**DOI:** 10.1186/s40798-021-00345-3

**Published:** 2021-08-25

**Authors:** Oliver R. Barley, Craig A. Harms

**Affiliations:** 1 Centre for Exercise and Sports Science Research, School of Medical and Health Sciences, Edith Cowan University, 270 Joondalup Drive, Joondalup, WA 6027 Australia; 2 School of Arts and Humanities, Psychology and Criminology, Edith Cowan University, Joondalup, WA Australia

**Keywords:** Martial arts, Training, Performance, Records

## Abstract

**Background:**

This study aimed to investigate the competitive history (the age when training and competing started), training habits and patterns of winning and losing of competitive combat sports athletes across different combat sports as well as the level of competition (e.g. amateurs, state-level and elite).

**Methods:**

Competitors (*N* = 298) from mixed martial arts (MMA), Muay Thai/kickboxing, boxing, Brazilian jiu-jitsu (BJJ), wrestling, judo and traditional striking sports (TSS) completed an online questionnaire.

**Results:**

Most athletes began competing in their mid-teenage years and competing soon after except for wrestlers who began earlier. Elite athletes began training earlier than amateurs (13.75 ± 7.75 years and 16.2 ± 7.45 years, *p*<0.01, respectively). Training habits were similar across sports (~4 combat and ~3 non-combat training sessions per week), except for MMA and wrestling which did more combat sports-specific training than Judo and TSS. Wrestlers did more non-combat sports-specific training than all other sports. Elite athletes completed more combat sessions per week than their lower-level contemporaries (4.64 ± 2.49 and 3.9 ± 1.44, *p*=0.01, respectively). Patterns of winning or losing were consistent across sports, except for amateur athletes who were more likely to report all their victories by points and none of their victories by submission or pin. Additionally, elite athletes are less likely to report none of their victories coming by knockout.

**Conclusions:**

Results may indicate that finishing ability is a key distinguisher of competitive level. The present study provides normative data for training and competing habits for athletes, support staff and regulators to use.

**Supplementary Information:**

The online version contains supplementary material available at 10.1186/s40798-021-00345-3.

## Key points

1. This study observed that training habits were largely similar across a range of combat sports with athletes completing ~4 combat and ~3 non-combat training sessions per week, except for MMA and wrestling which did more non-combat training sessions. The increased training load in MMA and wrestling may put those athletes at an increased risk of overtraining which should be monitored carefully by support staff.
2. It appears to be the case that ability to finish matches early (e.g. knock-out, pin, submission, etc.) is a distinguisher between the higher and lower level of competitions. Understanding the factors that are associated with a higher level may help with talent identification as well as tactical planning for competitions.
3. Most combat sports athletes began training at a similar age (14–16 years old), except for wrestlers who started training at a much younger age (~12 years old) which may have significant impacts on how regulators try to understand how athletes are recruited into the sports.

## Background

Combat sports are a collection of contact sports that typically involve one-on-one combat between competitors under a specific ruleset. The rulesets vary greatly between different combat sports, with techniques such as punches and kicks being utilised in striking sports, chokes and joint locks in grappling sports, and a mixture of both kinds of techniques in mixed-style sports [1]. Bouts can be concluded in many ways depending on the ruleset including finishes via the execution of specific techniques (e.g. pinning an opponent to the mat in wrestling), an opponent submitting or being rendered unconscious by a submission hold (e.g. joint manipulation or choke) or strike (e.g. punch or kick), official intervention (e.g. doctor determining it is unsafe to continue), and the totalling of points or a subjective judge’s decision [1]. The wide range of potential techniques and paths to victory result in diversity within competitions. Combat sports are popular worldwide with ~20% of gold medals being available in combat sports in the 2016 Olympic games and professional sports like boxing and mixed martial arts generating millions of views worldwide [2, 3]. Outside of elite-level competition, millions of members of the public worldwide practice combat sports recreationally [4, 5]. Despite this level of popularity, there are still significant gaps in the literature in profiling combat sports athletes. Such profiling research can be used to inform interventions both from a training and a policy perspective by informing governing bodies on the populations they aim to design rules and regulations for. Much of the previous research has focused on identifying physical characteristics (e.g. such as strength and power) relative to competitive success [6–8], with some research on the psychological profile (e.g. aggression and psychoticism) [9–12] of combat sports athletes.

While there is some research looking at training load in combat sports [13–16], there is a paucity of research examining the regular training habits of combat sports athletes across different disciplines. Kotarska and Nowak [17] found that most combat sports athletes trained 3–7 times a week, with the frequency being higher in competitive athletes. However, this study focused mostly on health-related factors as opposed to competitive ones (such as competitive records including different methods of victory). We are unaware of research that has investigated the training habits of combat sports athletes at different points in the competitive cycle (e.g. in regular training or prior to competition). While there is some research investigating the age that combat sports athletes began competing [18], there is a lack of research around deeper competitive histories including when athletes began to train. Some research has looked at common competitive outcomes, though much of it has focussed on outcomes more generally such as winning and losing relative to time-motion analysis [19] or how they are influenced by factors such as weight-cutting [20] and such data has not been reported in many combat sports. Further, little is known about the influence of factors such as the type of combat sports (e.g. boxing compared to wrestling) and current level of competition (e.g. professional and semi-professionals [elite] compared to amateurs) on training habits as well as competitive history and outcomes. For example, it is not known whether karate exponents began training and competing at different times compared to judo fighters, whether wrestlers are training more frequently before a competition than mixed martial arts fighters and whether elite combat sports athletes train more frequently in general and before a competition than amateur athletes.

The aim of this study was to examine training habits, competitive history and outcomes of combat sports athletes according to the type of combat sports and current level of competition. No hypotheses are proposed as the study is exploratory in nature. The potential role of age and body mass index (BMI) as potential covariates was examined to provide a comprehensive examination of these effects. BMI was selected due to the significant prevalence of weight manipulation within combat sports [18, 21, 22]. Developing a better understanding of the athletes competing within combat sports will allow competitors to identify the competitive styles and training habits associated with greater success as well as aiding organisers and regulators in developing better communication strategies.

## Methods

### Participants

Combat sports athletes that were at least 18 years old and registered in combat sports organisations and/or active members of combat sports gyms were recruited for this study. Participants were required to have a competitive record in a combat sports to be eligible for the study. Data was collected between August to December in 2019.

As can be seen in Table 1, most of the participants resided in the USA. In the previous 12 months, the participants had participated in a variety of martial arts including boxing, Brazilian jiu-jitsu (BJJ), Muay Thai/kickboxing (MT/KB), wrestling, mixed martial arts (MMA), judo, taekwondo, and karate. Given the low numbers in the Karate group, these participants were combined with the taekwondo to form a group for traditional striking sports (TSS: *n*=34/11.5%). About two thirds of the participants described themselves as either amateurs or competing at a regional or state level. The remaining approximately one third of the participants—National or International, Semi-Professional, or Professional—were combined into a single group of elite participants so that the participants in the present study could be categorised into three approximately equal groups.

**Table 1:** Summary of the participant’s background

Characteristic					Findings				
Country of residence	Total: *N* = 298/female: *N* = 42	USA: *n* = 145 (48.7%)/*n* = 11 (26.2%)	Australia: *n* = 43 (14.4%)/*n* = 12 (28.6%)	UK: *n* = 36 (12.1%)/*n* = 6 (14.3%)	Canada: *n* = 16 (5.4%)/*n* = 4 (9.5%)	Other: *n* = 58 (19.40%^a^)/ *n* = 9 (21%)			
Current primary sport	Total: *N* = 298/female: *N* = 42	Boxing: *n* = 61 (20.5%)/*n* = 8 (19%)	Brazilian jiu-jitsu: *n* = 58 (19.5%)/*n* = 6 (14.3%)	Muay Thai/kickboxing: *n* = 46 (15.4%)/*n* = 10 (23.8%)	Wrestling: *n* = 43 (14.4%)/*n* = 1 (2.4%)	Mixed Martial Arts: *n* = 30 (10.1%)/*n* = 5 (11.9%)	Judo: *n* = 26 (8.7%)/*n* = 1 (2.4%)	Taekwondo: *n* = 24 (8.1%)/*n* = 9 (21.4%)	Karate: *n* = 10 (3.4%)/*n* = 2 (4.8%)
Current level of competition	Total: *N* = 298/female: *N* = 42	Amateurs: *n* = 115 (38.6%) / *n* = 18 (42.9)	Regional/state: *n* = 99 (33.2%)/*n* = 6 (14.3%)	National or international: *n* = 56 (18.8%)/*n* = 14 (33.3%)	Semi-professional: *n* = 13 (4.4%)/*n* = 2 (4.7%)	Professional: *n* = 15 (5%)/*n* = 2 (4.7%)			

^a^In addition to the USA, Australia, the UK and Canada, participants resided in 33 other countries, with most countries represented by one to 6 participants

### Measures

Participants completed a questionnaire designed by the researchers which contains questions on personal information: their age, their sex (male or female), and the country in which they resided. The questionnaire also contained combat sports-specific questions, including height and weight (to calculate BMI); the age they began their involvement in combat sports; training habits, such as typical frequency of training for combat sports (typical and prior to competition), typical frequency of other forms of physical training (e.g. strength training); which combat sports they had competed in; which combat sports was their current primary combat sport; their highest level of competition (regional, state, national or international); self-reported competitive style (a visual analogue scale ranging from 0 to 100 with ‘defensive’ at 0 and ‘aggressive’ at 100); career stage (a visual analog scale ranging from 0 to 100 with ‘start of career’ at 0, ‘middle of career’ at 50 and ‘end of career’ at 100); and competitive outcomes, such as current competitive record (number of competitions, total wins, losses, draws/no contests) and methods of victory or loss (points or judge’s decision; knock-out, technical knock-out or corner/doctor’s; submission or pin; disqualification; Ippon, Waza-ari or technical fall) (Additional file 1).

### Procedure

Participants were informed of the study procedures and indicated their consent by accepting the terms and conditions before data collection. All experimental procedures are were approved by the Edith Cowan University’s Human Research Ethics Committee (Research Ethics Identification: 2019-00278-BARLEY). The participants completed the questionnaire in Qualtrics (Qualtrics August to December 2019, Qualtrics, Provo, Utah, USA), with the link to the survey being provided to the participants (either by direct e-mail or by advertisements posted on social media) by combat sports gyms and organisations around the world who agreed to help distribute the survey when contacted by the investigators. Several message boards where potential participants would visit were also identified and advertisements for the survey were posted where the relevant moderators approved for potential subjects to see and follow.

### Statistical Analysis

The preliminary analysis involved data screening (e.g. identification of outliers as well as examining the distribution of the data where relevant) and examining the correlation between age as well as BMI with the key study outcomes (competitive history and training habits as well as competitive style and competitive outcomes) to determine if age and BMI should act as covariates in the between-group analyses. The background (demographic background, current primary sport, current level of competition and self-reported stage of career) of the participants was reported. A one-way ANOVA was conducted to determine if the self-reported stage of career for the participants depended on the athlete’s primary sports as well as their current level of competition.

One-way ANOVAs were then conducted to determine if measures of competitive history and training habits and competitive outcomes depended on the current primary sports the participant engaged in and their current level of competition. Where significant main effects are observed, Bonferroni-adjusted post hoc tests were used to identify differences between groups. Alpha for these tests was set at .01 for the ANOVA tests and at .05 (because of the Bonferroni adjustment) for the post hoc tests. Cohen’s criteria [23] for effect size for *r* (≥ .1 = small, ≥ .3 medium, and ≥ .5 = large) and for *d* (≥ 0.2 = small, ≥ 0.5 = medium, and ≥ 0.8 = large) for the post hoc testing following ANOVA tests were applied in the present study. Interaction effects (that is, current primary sports x current level) were reported, but such effects were exploratory because of the uncertainty associated with such interaction effects. Alpha for these tests was also set at .01 for these tests.

Chi-square test of association was conducted to determine if self-reported patterns of winning and losing depended on the current primary sports the participants engaged in. The effect size was based on Pallant [24]. Some of the categories for this analysis were collapsed as some of the cells were initially found to have an expected count of less than 5 (see supplementary file). Analysis of self-reported patterns of winning and losing according to the current primary sports was not conducted due to many cells for each analysis where expected frequencies less than 5 were observed. Descriptive findings (M, SD, Pearson-*r* correlation, and frequency data) were reported as appropriate for each analysis. All analyses were performed using SPSS version 24 (SPAA Inc., Chicago IL, USA).

## Results

### Participant Characteristics

Two hundred ninety-eight individuals participated in the present study. Most of the participants were male (85.9%). As can be seen in Table 1, most of the participants resided in the USA, Australia, the UK and Canada. Most of the athletes participated in boxing or Brazilian jiu-jitsu, and the current level of competition of the participants was evenly spread between amateurs, regional and state, and elite (national or international, semi-professional and professional). Most of these findings were consistent for males and females except that, relatively speaking, very few female athletes from the sports of wrestling and judo or competing at a regional or state level participated in the study. In contrast, a relatively large number of female athletes for taekwondo and kickboxing as well as female athletes residing in Australia participated in the present study.

On average, the participants were 28.42 years (± 9.5) years of age, 1.76 m tall (± .09), weighed 78.73 kg (± 18.27), had a BMI of 25.32 (± 4.90) and were near the mid-point of their competitive career (VAS *M*= 45.71, ± 32.74). As can be seen in Table 2, the wrestlers were older than the MT/KB, boxing and TSS athletes. Further, the BMI of the participants did not depend on the participant’s current primary sport, nor their current competitive level. Finally, the wrestlers reported being at a later stage of their career than the MMA, MT/KB, boxing and BJJ athletes. Elite athletes were at a later stage of their career than the amateur athletes but not so compared to the regional/state athletes.

**Table 2:** Age, BMI and career stage: differences according to current primary sports and current level of competition

		*n*	Age (1)	BMI (2)		Stage of career (3)		
			*M*	*SD*	*M*	*SD*	*M*	*SD*
Current primary sport: *F*(6, 291) (1)= 3.69, *p*< .01, (2)*=* 1.88. *p*= .09; (3)= 9.08, *p*< .01.	MMA	30	27.73	10.70	25.78	5.88	27.93^a^	21.88
	MT/KB	46	27.30^a^	6.35	24.62	3.39	42.93^a^	33.53
	Boxing	61	25.92^a^	6.91	24.01	5.73	40.59^a^	30.53
	BJJ	58	29.38	7.96	25.43	4.10	38.91^a^	29.08
	Wrestling	43	**33.84**^**a**^	15.58	26.34	4.51	**74.56**^**a**^	30.06
	Judo	26	28.27	6.81	27.14	5.75	48.50^a^	30.47
	TSS	34	26.68^a^	7.37	25.33	4.74	47.35^a^	34.14
Current level of competition: *F*(2, 295) (1)= 1.02, *p*= .36; (2)= 2.71, *p* = .07; (3)= 6.43, *p*<.01.	Amateur	115	27.50	9.02	25.08	5.42	37.39^a^	32.47
	Regional/State	99	29.33	9.49	26.21	4.78	49.74	32.27
	Elite	84	28.61	10.03	24.60	4.14	**52.37**^**a**^	31.59

Note: *M* mean, *SD* standard deviation, *MMA* mixed martial arts, *MT/KB* Muay Thai/kickboxing, *BJJ* Brazilian jiu-jitsu and *TSS* traditional striking sports

Superscripts indicates where Bonferonni adjusted pair-wise tests indicated group differences, with scores in bold indicating the highest scores in a pairwise difference. Effect sizes for scores in bold. For age, wrestling and MT/KB (*d*= 0.55; medium effect); boxing (*d*= 0.63; medium effect) and TSS (*d*= 0.62; medium effect. For stage of career, wrestling and MMA (*d*= 1.85, large effect); MT/KB (*d*= 1.01, large effect); boxing (*d*= 1.13, large effect); BJJ (*d*= 1.21, large effect); judo (*d*= 0.87, large effect) and TSS (*d*= 0.85, large effect). Elite and amateur (*d*= 0.47, medium effect)

Two-way ANOVAs (current primary x current level: *F*(12, 277) were non-significant for (1) = 0.78, *p* = .68, (2)= 0.81, *p*= .64 and (3)= 0.80, *p*= .66

### Preliminary Analysis: Age, BMI and Study Variables

No univariate outliers or missing data were observed, and all values for normality for age, BMI, stage of career, competitive history, training habits and competitive style were within acceptable values (skew < 2 and kurtosis <7 [25]). As can be seen in Table 3, as age increased, so did BMI. Further, the age at which the participants began training and competing and competitive style was also positively associated with age. Higher BMI was associated with an earlier age when training and competing began and stage of their career. As the wrestlers were older and several of the correlations between the age of the participants were statistically significant, age was included as covariates for all inferential analyses. BMI was not included as a covariate in the analyses because it was associated with age and the correlations between BMI and other measures in the study were non-existent or small.

**Table 3:** Correlations between age, BMI, competitive history, training habits, competitive style and self-reported competitive outcomes

		Age	BMI
BMI		.29*^*^	1
Career stage		.50^**^	.25^*^
Competitive history	Age began training	.14*	−.13*
	Age began competing	.12*	−.14*
Training habits	How many times do train per week without a competition coming up? Combat sports sessions	−.08	−.05
	How many times do train per week without a competition coming up? Other forms of training (i.e. strength and conditioning)	.05	.07
	How many times do train per week with a competition coming up? Combat sports sessions	−.05	.01
	How many times do train per week with a competition coming up? Other forms of training (i.e. strength and conditioning)	.02	−.03
Winning and losing ratio	Winning ratio	−.03	−.05
	Losing ratio	.05	.05

Note: correlation was significant to .05* or .01 level**

### Competitive History

Overall, the participants had begun training before their 16th birthday (*M*= 15.56 years, ± 7.54) and began competing not long after their 18th birthday (*M*= 18.06 years, ± 7.50). As can be seen in Table 4, wrestlers started training at a younger age than boxing, MT/KB athletes and the BJJ athletes. Elite athletes began training at a younger age than the amateurs. Further, wrestlers began competing at a younger age than participants whose current primary sports was MMA, boxing, MT/KB, BJJ athletes and TSS athletes. Elite athletes began competing at an earlier age than the amateur athletes.

**Table 4:** Competitive history: differences according to current primary sports and current level of competition

		*n*	Age began training (1)		Age began competing (2)	
			*M*	*SD*	*M*	*SD*
Current primary sport: *F*(6, 290) (1)= 5.70, *p*< .01; (2)= 8.44, *p*< .01.	MMA	30	15.73	6.74	18.43^a^	5.84
	MT/KB	46	17.04^a^	6.58	19.91^a^	7.46
	Boxing	61	16.92^a^	6.64	19.79^a^	6.55
	BJJ	58	17.91^a^	8.13	20.47^a^	7.46
	Wrestling	43	**11.65**^**a**^	8.85	**12.65**^**a**^	8.85
	Judo	26	13.58	6.98	15.65	6.49
	TSS	34	13.38	6.19	16.74^a^	5.49
Current level of competition: *F*(2, 294) (1)= 6.25, *p* <.01; (2)= 5.75, *p*< .01.	Amateur	115	17.23^a^	6.42	19.62^a^	6.71
	Regional/state	99	15.14	8.21	17.84	8.08
	Elite	84	**13.75**^**a**^	7.75	**16.20**^**a**^	7.45

Note: *M* mean, *SD* standard deviation, *MMA* mixed martial arts, *MT/KB* Muay Thai/kickboxing, *BJJ* Brazilian jiu-jitsu and *TSS* traditional striking sports

Superscripts indicates where Bonferonni adjusted pair-wise tests indicated group differences, with scores in bold indicating the lowest score in the pairwise difference. Effect sizes for scores in bold. (1) Wrestling MT/KB (*d*= 0.70, medium effect), boxing (*d*= 0.66, medium effect) and BJJ (*d*= 0.74, medium effect). Elite and amateurs (*d*= 0.48, medium effect). (2) Wrestling and MMA (*d*= 0.81, large effect), MT/KB (*d*= 0.89, large effect), boxing (*d*= 0.88, large effect), BJJ (*d*= 0.98, large effect) and traditional striking sports (*d*= 0.58, medium effect). Elite and amateur (*d*= 0.48, medium effect)

Two-way ANOVAs (current primary x current level: *F*(12, 276) were non-significant for (1) = 1.03, *p*= .42; (2)= 1.20, *p*= .28

### Training Habits

Without a competition in the near term, the participants typically had approximately 4 (*M*= 4.12 ± 1.83) combat training sessions per week and 3 (*M*=2.99 ± 2.00) sessions for other forms of training such as strength and condition training. As noted in Table 5, MMA athletes reported doing more combat sports training sessions per week without a competition in the near term than the judo and TSS athletes, wrestling did more such training than judo and TSS, and BJJ did more such training than TSS. Elite athletes did more training than did amateurs and region/state athletes. Further, wrestlers doing more other forms of training each week without a competition in the near term than did MMA, MT/KB, BJJ, judo or TSS athletes.

**Table 5:** Typical frequency training (combat sports and other sessions) without a competition coming up: differences according to current primary sports and current level of competition

		*n*	Combat sports sessions (1)		Other sessions (2)	
			*M*	*SD*	*M*	*SD*
Current primary sport: *F*(6, 290) (1)= 5.52, *p*< .01; (2)= 6.91, *p*< .01.	MMA	30	**4.90**^**a**^	1.61	3.00^a^	2.21
	MT/KB	46	4.41	2.04	2.59^a^	1.67
	Boxing	61	3.80	1.55	3.18	2.23
	BJJ	58	**4.41**^**c**^	2.04	2.52^a^	1.38
	Wrestling	43	**4.58**^**b**^	2.05	**4.58**^**a**^	2.25
	Judo	26	3.15^ab^	1.26	2.65^a^	1.72
	TSS	34	3.24^a,bc^	1.18	2.26^a^	1.60
Current level of competition: *F* (2, 294) (1)= 5.01, *p*= .01; (2)= 0.89, *p*= .42.	Amateur	115	3.90^a^	1.44	3.06	2.11
	Regional/State	99	3.92^a^	1.49	2.79	1.72
	Elite	84	**4.64**^**a**^	2.49	3.14	2.18

Note: *M* mean, *SD* standard deviation, *MMA* mixed martial arts, *MT/KB* Muay Thai/kickboxing, *BJJ* Brazilian jiu-jitsu and *TSS* traditional striking sports

Superscripts indicates where Bonferonni adjusted pair-wise tests indicated group differences, with scores in bold indicating the highest scores in pairwise differences. (1) MMA and judo (*d*= 1.24, large effect) and TSS (*d*= 1.18, large effect). Wrestling and judo (*d*= 0.91, large effect) and TSS (*d*= 0.84, large effect). BJJ and judo (d= 0.83, large effect) and TSS (*d*= 0.76, large effect). Elite and amateur (*d*= 0.35, small effect) and regional/state (*d*= 0.35, small effect). (2) Wrestling and MMA (*d*= 0.70, medium effect), MT/KB (*d*= 1.01, large effect), boxing (*d*= 0.63, medium effect), BJJ (*d*= 1.08, large effect), judo (*d*= 1.01, large effect) and TSS (*d*= 1.23, large effect)

Two-way ANOVAs (current primary x current level: *F*(12, 275) were non-significant for (1) = 1.07, *p*= .39; (2) = 0.63, *p*= .81

Without a specific competition coming up to train for, the participants typically completed 5 (*M*= 5.20 ± 1.92) combat training sessions per week and 3 (*M*= 3.30 ± 2.15) sessions for other forms of training such as strength and condition training. As noted in Table 6, MMA athletes did more combat sports sessions per week than TSS and judo athletes, MT/KB did more such training than did judo and TSS, BJJ did more than TSS, and wrestlers did more than judo and TSS athletes. Further, the wrestlers reported doing more other forms of training each week without a competition in the near term than did the judo, or TSS athletes.

**Table 6:** Typical frequency of training (combat sports and other sessions) with a competition coming up: differences according to current primary sports and current level of competition

		*n*	Combat sports sessions (1)		Other sessions (2)	
			*M*	*SD*	*M*	*SD*
Current primary sport: *F*(6, 290) (1)= 6.21, *p*< .01; (2)= 4.31, *p* <.01.	MMA	30	**6.37**^**a**^	2.03	3.23	2.24
	MT/KB	46	**6.02**^**b**^	2.40	3.28	1.97
	Boxing	61	5.55	2.30	3.75	1.94
	BJJ	58	**5.21**^**c**^	1.46	2.72^a^	1.54
	Wrestling	43	**5.98**^**d**^	2.87	**4.42**^**a**^	2.50
	Judo	26	4.15^abd^	1.62	2.73	1.73
	TSS	34	3.91^abcd^	1.22	2.56^a^	2.72
Current level of competition: *F* (2, 294) (1)= 1.85, *p*= .16; (2)= 0.95, *p*= .39.	Amateur	115	5.30	1.84	3.33	2.13
	Regional/State	99	5.17	2.11	3.08	1.83
	Elite	84	5.81	3.10	3.51	2.51

Note: *M* mean, *SD* standard deviation, *MMA* mixed martial arts, *MT/KB* Muay Thai/kickboxing, *BJJ* Brazilian jiu-jitsu and *TSS* traditional striking sports

Superscripts indicates where Bonferonni adjusted pair-wise tests indicated group differences, with scores in bold indicating the highest scores in pairwise differences. (1) MMA and judo (*d*= 1.24, large effect) and TSS (*d*= 1.47, large effect). MT/KB and judo (*d*= 0.98, large effect) and TSS (d= 1.18, large effect). BJJ and TSS (*d*= 1.00, large effect). Wrestling and judo (*d*= 0.85, large effect) and TSS (*d*= 0.99, large effect). (2) Wrestling and MMA (*d*= 0.51, medium effect), MT/KB (*d*= 0.51, medium effect), boxing (*d=*0.30, small effect), BJJ (*d*= 0.80, large effect); judo (*d*= 0.84, large effect) and TSS (*d*= 0.72, medium effect)

Two-way ANOVAs (current primary x current level: *F*(12, 276) were non-significant for (1)= 1.75, *p*= .07; (2)= 0.59, *p*= .85

### Patterns of Winning or Losing and Current Level of Competition (see Tables 7 and 8)

The pattern of victories and losses according to points or judge’s decision was associated with the current level of competition. The proportion of amateurs that won all of their contests by points or judge’s decision was greater than expected whereas the proportion of elite athletes who won all of their contests by points of judge’s decisions was less than expected. The proportion of amateurs who lost some of their contests by points or judge’s decision was less than expected whereas the proportion of regional/state athletes who lost some of their contests by points or judge’s decision was greater than expected.

**Table 7:** Self-reported winning and losing ratios: differences according to current primary sports and current level of competition

		*n*	Wining ratio (1)		Losing ratio (2)	
			*M*	*SD*	*M*	*SD*
Current primary sport: *F* (6, 290): (1)= 1.34, *p*= .24; (2) 1.36, *p*= .23.	MMA	30	65.10	29.36	29.30	28.11
	MT/KB	46	62.73	27.55	31.44	23.94
	Boxing	61	64.99	30.12	29.99	27.29
	BJJ	58	61.52	23.55	34.90	22.57
	Wrestling	43	69.89	19.19	30.03	19.25
	Judo	26	61.80	21.20	37.69	21.28
	TSS	34	54.30	30.97	41.82	31.17
Current level of competition: *F* (2, 294): (1)= 1.41, *p*= .25; (2)= 1.92, *p*= .15.	Amateur	115	61.82	30.00	31.43	26.56
	Regional/state	99	61.30	25.24	37.23	25.14
	Elite	84	67.28	22.45	30.61	22.32

Notes: *M* mean, *SD* standard deviation, *MMA* mixed martial arts, *MT/KB* Muay Thai/kickboxing, *BJJ* Brazilian jiu-jitsu, *TSS* traditional striking sports

Superscripts indicate where Bonferonni adjusted pair-wise tests indicated group differences, with scores in bold indicating the lowest scores in pairwise differences. (1) TSS and boxing (*d*= 0.67, medium effect), MT/KB (*d*= 0.83. large effect) and MMA (*d*= 0.90, large effect)

Two-way ANOVAs (current primary x current level: *F*(12, 276) were non-significant for (1)= 0.61, *p*= .83; (2)= .65, *p*= 80

**Table 8:** Frequency of self-reported types of wins and losses according to the current level of competition

			None	Few	Some	Most	All	Total
			O/E	O/E	O/E	O/E	O/E	
Points or judge’s decision	Victories: χ^2^ (8, *n*= 282)= 30.58, *p*< .01, Cramer’s V = .23 (medium effect)	Amateur	20/14.7	12/17.5	16/24.2	27/30.6	**37**^a^/25	112
		Regional/state	11/11.7	14/13.9	27/19.3	19/24.3	18/19.9	89
		Elite	6/10.6	18/12.6	18/17.5	31/22.1	**8**^a^/18.1	81
		Total	37	44	61	77	63	282
	Losses: χ^2^ (8, n= 282)= 18.33, p< .02, Cramer’s V = .18 (medium effect)	Amateur	33/24.6	11/12.3	**6**^a^/14.3	23/28.2	39/32.6	112
		Regional/state	13/19.6	11/9.8	**18**^a^/11.4	24/22.4	23/25.9	89
		Elite	16/17.8	9/8.9	12/10.3	24/20.4	20/23.6	81
		Total	62	31	36	71	82	282
Knockout			None	Few	Some	Most or All		Total
			O/E	O/E	O/E	O/E		O/E
	Victories: χ^2^ (6, *n*= 201)= 17.70, *p*< .01, Cramer’s V = .21 (medium effect).	Amateur	43/41.2	17/21.3	12/14.9	19/13.6		91
		Regional/state	32/24.4	10/12.6	9/8.9	3/8.1		54
		Elite	16/25.4^a^	20^a^/13.1	12/9.2	8/8.4		56
		Total	91	47	33	30		201
			None	Few	Some	Most or all		Total
			O/E	O/E	O/E	O/E		O/E
Submission or pin	Victories: χ^2^ (6, *n*= 183)= 15.87, *p*= .01, Cramer’s V = .21 (medium effect).	Amateur	**20**^a^/11.5	8/7	13/18.2	8/12.3		49
		Regional/state	15/18.3	8/11.1	30/29	25/19.6		78
		Elite	8/13.2	10/8	25/20.8	13/14.1		56
		Total	43	26	68	46		183
	Losses: χ^2^ (6, *n*= 183)= 7.26, *p*= .30.	Amateur	22/16.1	14/15.5	6/9.6	7/7.8		49
		Regional/state	21/25.6	23/24.7	19/15.3	15/12.4		78
		Elite	17/18.4	21/17.7	11/11.0	7/8.9		56
		Total	60	58	36	29		183
Ippon, Waza-ari or technical fall			None	Not none				Total
			O/E	O/E				
	Victories: χ^2^ (2, *n*= 117)= 10.03, *p*= .01, Cramer’s V = .29 (medium effect).	Amateur	**19**^a^/12.3	11/11.7				30
		Regional/state	19/19.7	29/28.3				48
		Elite	10/16.0	29/23.0				39
		Total	48	69				117
	Losses: χ^2^ (2, *n*= 117)= 9.1, *p*= .01.	Amateur	23/15.9	**7**^a^/14.1				30
		Regional/state	22/25.4	26/22.6				48
		Elite	17/20.7	22/18.3				39
		Total	62	55				117

*Note*: *O* observed count, *E* expected count. ^a^ and bolded = standardized residual > 1.96. Points or judge’s decision applies to all sports. Knockout applies to Muay Thai/kickboxing, mixed martial arts and traditional striking sports. Submission or pin applied to Brazilian jiu-jitsu, wrestling, mixed martial arts and judo. Ippon, Waza-ari or technical fall applies to judo and wrestling

For wins by knockout, and after combining the ‘most’ and ‘all’ categories, the pattern of winning was associated with the current level of competition. The only difference between levels of competition that approached statistical significance was that the proportion of elite athletes winning no contests by knockout was less than expected and the proportion of elite athletes winning a few contests by knockout was more than expected. Patterns of losses according to knockout and according to the level of competition were not examined because three quarters of the participants (75.6%) reported never having a loss by knockout.

For wins by submission or pin, and after combining the ‘most’ and ‘all’ categories, the pattern of winning (but not losing) was associated with the current level of competition. The proportion of amateur athletes who reported never winning by submission or pin was greater than expected. Chi-square tests of association for winning or losing by disqualification were not conducted as 80% of all participants had neither experienced a victory (189 of 226 respondents) or a loss (201 of 226 respondents) by disqualification.

For Ippon, Waza-ari or technical fall, and after retaining two categories of winning and losing by this method, this pattern of winning and losing was associated with the current level of competition. The only difference that approached statistical significance was that the proportion of amateur athletes who reported never winning by this method was less than expected. The only difference between levels of competition that approached statistical significance was that the proportion of amateur athletes who reported never losing by this method was less than expected.

## Discussion

This study sought to describe the competitive history (i.e. age at which training and competing began), their training habits (combat sports and other sessions; typical and before a competition) and competitive outcomes of combat sports athletes and to determine if the competitive history, training habits and competitive outcomes depended on the types of combat sports and current level of competition of the combat sports athletes. The average age of athletes was also similar across sports, with most athletes being in their mid to late twenties, and the only observed difference was the traditional striking athletes which were slightly younger than their contemporaries.

The competitive histories of athletes were explored within the present study, and some interesting findings between sports were observed. The age that the participants began training and competing was similar across all combat sports except for wrestlers, who started both training and competing significantly earlier than all other sports questioned as well as being closer to the end of their career than most other combat sports. It is possible that there may be a difference in the culture around wrestling compared with other combat sports where children are encouraged to begin training and competing in wrestling at a relatively young age. This finding may also be influenced by the number of participants in the present study from the USA where there is a scholastic folkstyle wrestling program across middle schools, high schools and universities with millions of active participants each year [18, 26]. We also found that the higher-level athletes began both training and competing earlier than both the lower levels of competition (amateur and regional/state). The results of the present study align with previous research that found competitive athletes to be younger than their non-competitive contemporaries [17]. It may be the case that starting both training and competing earlier is associated with reaching a higher competitive level. Indeed, previous research has observed success in competitions at a youth level to be a predictor or future success at senior competition in taekwondo, wrestling and boxing [27]. Future research should further look to investigate the potential influences of the age training and competing begins on long-term wellbeing and competitive success to help maximise the health and success of combat sports athletes.

This study collected much information on the training habits of combat sports athletes. The frequency of training was similar across most combat sports with most athletes completing ~4–5 combat and ~2–3 non-combat sessions per week without a competition coming up and the frequency of combat sessions increasing when a competition is coming up. These results are comparable to previous research that observed that most competitive combat sports athletes trained 3–7 times a week [17]. When comparing between combat sports, we observed that MMA athletes generally completed more combat sessions than other sports both with and without a competition coming up, which may be a result of the wider range of skills (both striking and grappling) that they must practice. It was also found that elite athletes had more combat sessions per week than their lower-level contemporaries (amateur and regional/state), which would plausibly be the result of an increased commitment to training. Though interestingly, there was no difference between competitive levels and the frequency of non-combat sessions, which may suggest that the development of combat sports-specific skills is more important for competitive success than general strength and conditioning. The level of planning that goes into the regular training of the athletes that took part in this study and across combat sports in general is unclear. While there is a body of research looking at optimal loading and periodisation in combat sports [14–16, 28, 29], it may be the case (especially at a lower level of competition) that such information is underutilised by competitors and support staff, which future research should aim to investigate.

There was a diverse range of competitive outcomes observed within the present study, yet no significant differences in winning or losing ratio between combat sports or competitive level were observed within the present study. However, we did observe that more amateur athletes than expected win all their victories via points or judge’s decision and whereas fewer than expected elite athletes reported that all of their victories came using this method. We also observed more regional/state athletes reporting some of their losses to come by points or judge’s decision than expected but less than expected in amateur athletes. More amateur athletes than expected reported none of few of their wins coming by submission or pin as well as Ippon, Waza-ari or technical fall. Additionally, we observed fewer elite athletes reporting none of their wins coming by knockout than expected. The higher finishing rate in elite athletes combined with the increased rate of bouts decided by judges or points in amateurs may indicate that finishing ability is a key distinguisher between lower and higher-level competitive levels. However, it is worth noting that we did observe more elite athletes reporting only a few of their victories to come by knockout than expected which would contradict the rest of the trends within our data. Interestingly, while previous research has examined the factors underpinning competitive outcomes in combat sports [19, 30], there is a lack of research examining the differences between different competitive levels from an outcomes perspective. Much of the previous research comparing competitive levels in combat sports has focused on physiological and physical attributes [7, 31, 32] instead of competitive outcomes. Better understanding such factors could aid in talent identification as well as tactical planning for athletes and support staff.

The present study analysed the potential correlations of age and BMI within the present study. Small but consistent findings about the association between age and many of the variables in the present study indicate that researchers should consider age to a covariate in analyses that involve research with combat sports athletes. A small positive correlation was observed between age and BMI, as well as a moderate correlation with career state. Logically, it does stand to reason that older age would be associated with increased body mass and being in a later state of a sporting career. The participants in this study typically had a BMI between 24 and 27, and there were no significant differences between combat sports which is surprising considering the higher prevalence of rapid weight loss strategies in some combat sports compared to others, such as MMA [18]. A small positive correlation was observed between BMI and career stage, which could be related to the increased age that people later in their career are likely to be. Given the vast majority of findings regarding BMI were not significant or of negligible strength, future research may not need to carefully control for BMI.

This study had many strengths including the large sample of athletes questioned as well as the in-depth questionnaire utilised. However, there are some limitations which must be considered. There are potentially issues with relying on self-reported data, though a sufficiently large total athlete sample was recruited to try and mitigate this confounder. Regardless, the potential limitation of the data being self-reported must be considered when interpreting the results. Unfortunately, there was not a large enough female sample to examine potential sex-based differences in combat sports profiles. While age was accounted for, there would be value in replicating the current study with wrestlers who were younger and at an earlier stage of their career, considering that the wrestlers in the present study reported being closer to the end of their career. Around half of the participants in this study were from the USA, there may be a benefit in conducting specific investigations of athletes in different countries to example potential differences across cultures. It is also worth noting that the questionnaire was only available in English so it is unclear if the results would apply to non-English speaking populations. There would also be value in the future of getting a larger sample of karate and taekwondo competitors, as in the current study that had to be combined into the traditional striking sports category for analysis.

## Conclusion

This study provides important information on the competitive habits/outcomes and history of combat sports athletes as well as highlighting the general homogeneity of findings across different types of combat sports as well as different levels of competition. Such information can be used to understand the developmental trajectories of combat sports participants as well as providing guidance regarding benchmarks for BMI, typical training schedules and competitive outcomes across different combat sports and competitive levels. These benchmarks can help inform training program design as well as the development of policies by regulatory bodies and organisers. For example, a better understanding of training frequency can help inform advice given on injury prevention, especially across different combat sports (different advice may need to be given to MMA athletes than those in BJJ). Future research should aim to further examine how differing factors (e.g. training frequency and age begun competing) relate to competitive success across competitive levels.

## Supplementary Information

**Additional file 1.** Frequency of self-reported wins and losses by knock-out according to current level of competition: Initial frequencies. Frequency of self-reported wins and losses by submission or pin according to current level of competition: Initial frequencies. Frequency of self-reported wins and losses by Ippon, Waza-ari or technical fall according to current level of competition: Initial frequencies. Questionnaire: *Note: This questionnaire was delivered in Qualtrics, so the below serves as a description of the survey*.

## Data Availability

The datasets generated and/or analysed during the current study are not publicly available due to ethical restraints but are available from the corresponding author on reasonable request.

## Abbreviations

BMI: Body mass index
BJJ: Brazilian jiu-jitsu
MT/KB: Muay Thai/kickboxing
MMA: Mixed martial arts
TSS: Traditional striking sports
