# Supplementary material for: Profiling Combat Sports Athletes: Competitive History and Outcomes According to Sports Type and Current Level of Competition
Source: Sports Med Open. 2021 Aug 25;7:63. doi: 10.1186/s40798-021-00345-3 (PMC8387537; doi:10.1186/s40798-021-00345-3)
Supplement: Supplementary file 1 — Additional file 1. Frequency of self-reported wins and losses by knock-out according to current level of competition: Initial frequencies. Frequency of self-reported wins and losses by submission or pin according to current level of competition: Initial frequencies. Frequency of self-reported wins and losses by Ippon, Waza-ari or technical fall according to current level of competition: Initial frequencies. Questionnaire: *Note: This questionnaire was delivered in Qualtrics, so the below serves as a description of the survey*. [file 40798_2021_345_MOESM1_ESM.docx]

Supplementary file

Article title: Profiling Combat Sports Athletes: Competitive History and Outcomes According to Sport Type and Current Level of Competition

Journal name: Sports Medicine – Open

Author names: Oliver R. Barley^1^ & Craig A. Harms^2^

Author affiliations

^1^ Centre for Exercise and Sports Science Research, School of Medical and Health Sciences, Edith Cowan University, Joondalup, WA, Australia

^2^ School of Arts and Humanities, Psychology and Criminology, Edith Cowan University, Joondalup, WA, Australia

Email address of corresponding author: [o.barley@ecu.edu.au](mailto:o.barley@ecu.edu.au)

*Frequency of self-reported wins and losses by knock-out according to current level of competition: Initial frequencies*

|  | None | Few | Some | Most | All | Total |
| --- | --- | --- | --- | --- | --- | --- |
| Victories | 91 | 47 | 33 | 17 | 13 | 201 |
| Losses | 152 | 30 | 6 | 5 | 8 | 201 |

*Frequency of self-reported wins and losses by submission or pin according to current level of competition: Initial frequencies*

|  | None | Few | Some | Most | All | Total |
| --- | --- | --- | --- | --- | --- | --- |
| Victories | 43 | 26 | 68 | 35 | 11 | 183 |
| Losses | 60 | 58 | 36 | 16 | 13 | 183 |

*Frequency of self-reported wins and losses by Ippon, Waza-ari or technical fall according to current level of competition: Initial frequencies*

|  | None | Few | Some | Most | All | Total |
| --- | --- | --- | --- | --- | --- | --- |
| Victories | 48 | 17 | 28 | 22 | 2 | 117 |
| Losses | 62 | 22 | 13 | 17 | 3 | 117 |

**Questionnaire**

****Note: This questionnaire was delivered in Qualtrics, so the below serves as a description of the survey****

Q1. What year were you born?

*Dropdown list from 1920-2001*

Q2. What is your sex?

*Male or female*

Q3. How tall are you? (Please specify your unit of measurement such as centimetres or feet)

*Text entry*

Q4. How much do you currently weigh? (Please specify your unit of measurement such as kilograms or pounds)

*Text entry*

***Page break***

Q5. At what age did you begin training combat sports? (Years)

*Dropdown list from 4-70*

Q6. At what age did you begin to compete in combat sports? (Years)

*Dropdown list from 4-70*

Q7. How many times do you train per week without a competition coming up?

Two d*ropdown lists (first is combat sports sessions and second is other forms of training (i.e. strength and conditioning) scale points range from 1-14+*

Q8. How many times do you train per week with a competition coming up?

Two d*ropdown lists (first is combat sports sessions and second is other forms of training (i.e. strength and conditioning) scale points range from 1-14+*

***Page break***

Q9. Which of the previous combat sports have you previously competed in? (Check all that apply)

*Multi-choice matrix with Mixed Martial Arts, Muay Thai/Kickboxing, Boxing, Brazilian Jiu Jitsu, Wrestling, Judo, Karate or Thai Kwon Do.*

Q10. Which one of these combat sports is your current primary combat sport? (i.e. the one most of your training within the last 12 months was in preparation for)

*Multi-choice (only can be checked) matrix with Mixed Martial Arts, Muay Thai/Kickboxing, Boxing, Brazilian Jiu Jitsu, Wrestling, Judo, Karate or Thai Kwon Do.*

***Page break***

S1. Please answer all following questions for your previously indicated primary combat sport only.

**Page break**

Q11. Please describe what levels of competition you have competed at? (Note: some levels may not be applicable to your sport, in such a case, please enter "never participated")

*Matrix table with competitive levels (Regional/state level, National/international level, amateur, semi-professional and professional). Scale points are participated, for a championship and never participated.*

Q12. Which one of these competitive levels is your current primary level of competition? (i.e. the one most of your training within the last 12 months was in preparation for)

*Multi-choice single answer with Regional/state level, National/international level, amateur, semi-professional and professional.*

**Page break**

S2. Please answer all following questions for your primary level of competition only.

**Page break**

Q13. At what stage are you in your competitive career? (please estimate)

*A visual analogue scale ranging from 0-100 with “start of career” at 0, “middle of career” at 50 and “end of career” at 100.*

Q14. Please describe your current competitive record (If unsure, please estimate).

*Dropdown Likert scale with “Wins (total)”, “Losses (total) and “Draws or no contests (total)”. Ranging from 0-300 and 300+*

Q15. How would you describe your competitive style?

*A visual analogue scale ranging from 0-100 with “Defensive” at 0 and “aggressive” at 100.*

Q16. Please describe number of victories you have had by different methods (If the method of victory does not apply to your sport, please select "not applicable")

*Dropdown Likert scale with points or judge’s decision, Knock-out, technical knock-out or corners/doctor’s stoppage, submission or pin/technical fall, disqualification, ippon or Waza-ari. Scale includes all my victories, most of my victories, some of my victories, a few of my victories, none of my victories and not applicable.*

Q17. Please describe number of losses you have had by different methods (If the method of loss does not apply to your sport, please select "not applicable")

*Dropdown Likert scale with points or judge’s decision, Knock-out, technical knock-out or corners/doctor’s stoppage, submission or pin/technical fall, disqualification, ippon or Waza-ari. Scale includes all my losses, most of my losses, some of my losses, a few of my losses, none of my losses and not applicable.*
